# Bernese Periacetabular Osteotomy in a Hip Extra-Articular Resection Followed by Reconstruction Using an Extracorporeal Irradiated Acetabulum Autograft with Megaprosthesis, for Proximal Femur Osteosarcoma in a Pediatric Patient

**DOI:** 10.1155/2015/813683

**Published:** 2015-06-25

**Authors:** Victor Barro, Roberto Velez, Daniel Pacha, Jordi Giralt, Isabel Roca, Marius Aguirre

**Affiliations:** ^1^Orthopaedic Surgery Department, Vall d'Hebron Hospital, 08035 Barcelona, Spain; ^2^Radiation Oncology Department, Vall d'Hebron Hospital, 08035 Barcelona, Spain; ^3^Nuclear Medicine, Department of Radiology, Vall d'Hebron Hospital, 08035 Barcelona, Spain

## Abstract

Osteosarcoma is the most common primary malignant tumour of bone. The oncologic surgery of a proximal femur osteosarcoma affecting the hip joint can be very challenging. We present an 8-year-old boy with a 5-month history of right hip pain. Radiographs and magnetic resonance imaging (MRI) showed a lytic lesion of the proximal femur extending 13 cm to the diaphysis. Histological evaluation was consistent with high-grade osteoblastic osteosarcoma. After completing chemotherapy we performed an extra-articular resection of the hip. Reconstruction was accomplished by reimplanting the acetabulum after irradiation and modular proximal femur megaprosthesis. Endoprosthetic reconstruction following proximal femur resection is a good treatment alternative achieving good postoperative function. Extra-articular resection of the hip using a periacetabular osteotomy technique enabled us to achieve wide margins and leave an intact posterior column to optimize acetabular reconstruction stability. Extracorporeal irradiation and reimplantation is a valuable treatment option in a situation such as this where allograft geometric fit is a priority. We conclude that an extra-articular resection of the hip, followed by reconstruction with an extracorporeally irradiated acetabulum and a proximal femur modular megaprosthesis, is a useful combined treatment option for malignant lesions involving the hip joint, especially in paediatric patients.

## 1. Introduction

Osteosarcoma is the most common primary malignant tumor of bone, with an incidence of 2.4% among childhood cancers [1]. Osteosarcoma commonly occurs in the long bones, near the metaphyseal growth plates; the femur is the most frequently involved bone. Articular cartilage acts as a relative barrier to tumor spread, and extension into the hip joint is extremely rare [2].

The management of a proximal femur malignant lesion with intra-articular involvement is challenging. The need for adequate excision margins and the anatomical complexity of the pelvis mean that reconstruction procedures are associated with high rates of morbidity and mortality, as well as poor functional outcome [3].

We present an unreported case of proximal femur osteosarcoma with intra-articular involvement, treated with extra-articular resection of the hip, reconstructed with an irradiated acetabulum structural autograft and proximal femur modular megaprosthesis replacement.

## 2. Case Report

**Figure 1:**
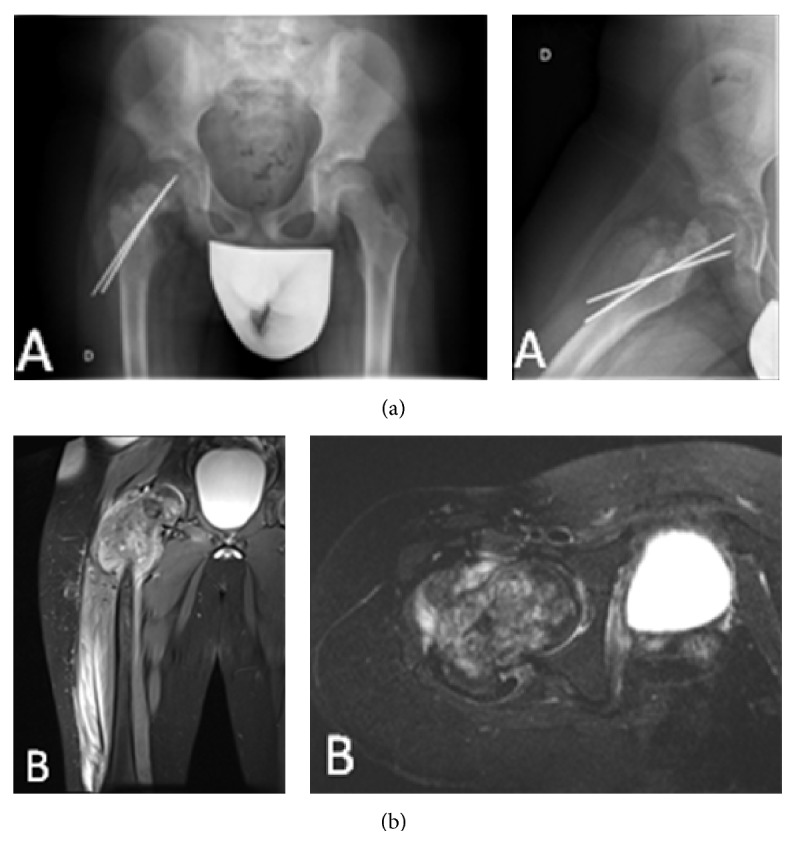
(a) Plain radiographs of the pelvis, hip, and proximal femur showing mixed osteolytic and sclerotic areas in the right proximal femur and an unhealed femoral neck fracture fixed with two K wires. (b) MRI showing a proximal femoral lesion extending distally 13 cm with a surrounding soft-tissue mass.

We present a case of a previously healthy 8-year-old boy with a 5-month history of intermittent right hip and knee pain. The patient suffered a nondisplaced pathological femoral neck fracture, treated at another centre with closed reduction and internal fixation using two K wires. After two months of follow-up without clinical or radiological signs of fracture healing, the patient was referred to our centre. Plain radiographs of the pelvis, hip, and proximal femur showed mixed osteolytic and sclerotic areas at the right proximal femur and an unhealed femoral neck fracture fixed with two K wires (Figure 1(a)). Suspecting a primary bone tumor, an open biopsy was performed and the K wires were removed. Initial magnetic resonance imaging (MRI) showed a proximal femur lesion extending distally 13 cm, with a surrounding soft-tissue mass. MRI also showed joint effusion, though there was no evidence of acetabular extension (Figure 1(b)). Histological evaluation was consistent with high-grade osteoblastic osteosarcoma. A chest CT scan and a bone scintigraphy did not show metastasis. Neoadjuvant chemotherapy with the Erasmus 1 Trial protocol (Methotrexate, Adriamycin, and Cisplatin) was initiated and completed without incident.

With the patient in supine position, a utilitarian skin incision, as described by Kabukcuoglu et al. [4], was used. The iliac vessels and the femoral and sciatic nerves were exposed and mobilized. The intrapelvic portion of the psoas muscle was released and the gluteus maximus muscle was detached from the iliotibial band and femur. The external rotators of the hips were transected near their origins, and the vastus intermedius and lateralis were transected at the level of the femoral osteotomy.

**Figure 2:**
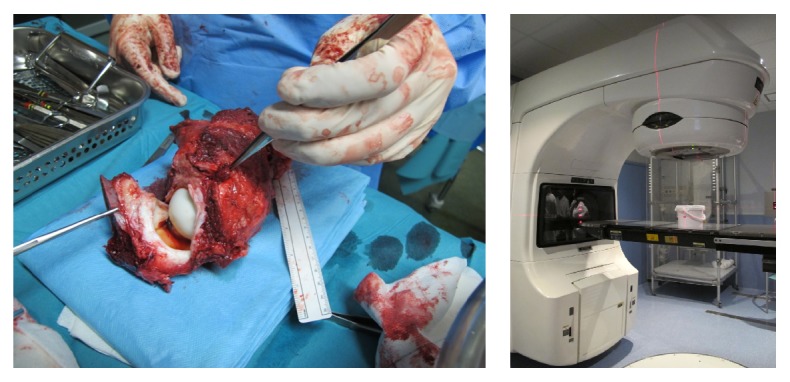
Following the Bernese periacetabular osteotomy, an en bloc extra-articular resection of the acetabulum was performed; the resected specimen was then transferred to a separate sterile trolley. A capsulectomy was made through the acetabular rim, and the articular fluid and proximal femur were sent for histological examination. The acetabulum was placed in a sterile container and sent to the radiotherapy department for extracorporeal irradiation.

A femoral osteotomy was made 16 cm distally from the tip of the greater trochanter, giving 3 cm of wide margin. The insertions of the adductors were transected from the proximal femur, followed by release of the gluteus medius and minimus. Subsequently an en bloc extra-articular resection of the acetabulum was performed, following the standard steps in a Bernese periacetabular osteotomy; the resected specimen was then transferred to a separate sterile trolley and moved away from the main operative field to avoid any contamination. Under aseptic precautions, a capsulectomy was made through the acetabulum rim, and samples of articular fluid and the proximal femur were sent for histological examination. Head size was measured for bipolar reconstruction. The acetabulum was placed in a sterile container and sent to the radiotherapy department for extracorporeal irradiation (Figure 2). The acetabulum segment, enclosed in a sterile pack, was irradiated to a dose of 50 Gy/1 fraction. Meanwhile we implanted a bipolar femoral modular megaprosthesis (Biomet OSS Compress) for proximal femoral reconstruction. Upon return to the operating room, the excised acetabulum was reimplanted and fixed with two reconstruction plates, achieving perfect geometric anatomic fit.

Histological analysis revealed wide margin resection with 95% necrosis of the osteogenic osteosarcoma. Microscopic tumor implantations were seen at the distal capsular insertions. Articular fluid cytology showed no evidence of tumor cells.

**Figure 3:**
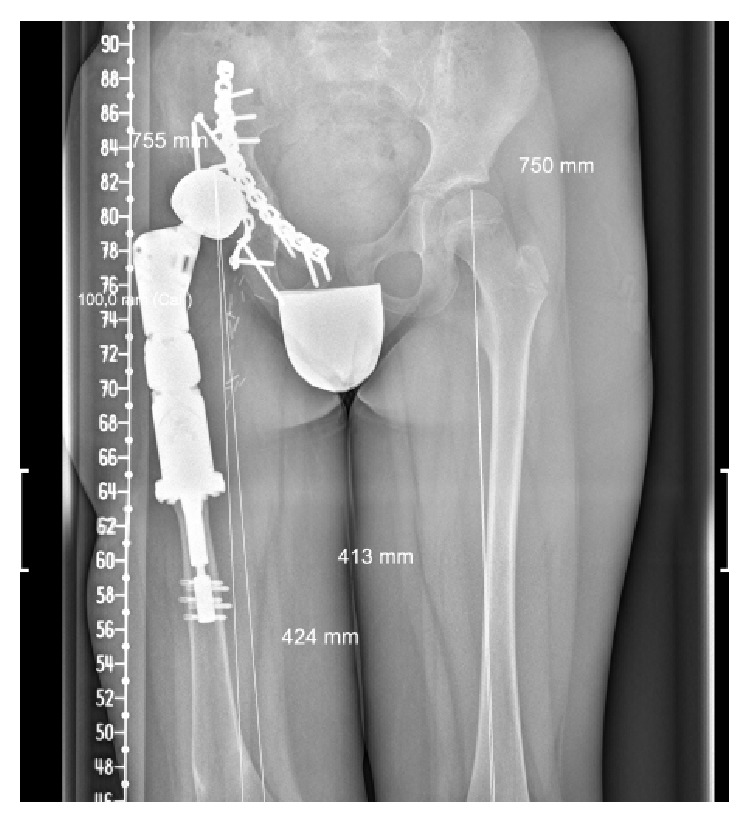
After 18 months of follow-up, a plain AP radiograph of the pelvis shows correct integration of the autograft and prosthesis.

Postoperatively the patient was immobilized with a spica cast for a period of 12 weeks. Three weeks after the index procedure the patient developed a superficial wound dehiscence which required aggressive surgical debridement and lavage. Tissue cultures were positive for* E. coli*. The patient received a 6-week course of intravenous Ciprofloxacin followed by 12 weeks of oral Ciprofloxacin. The patient initiated non-weight-bearing ambulation at 6 weeks. Three months after surgery the patient was allowed to progressively bear weight. Radiographic follow-up showed correct integration of the autograft and prosthesis (Figure 3). Currently, after 18 months, the patient walks with the use of a cane and there is no evidence of infection or tumor recurrence.

## 3. Discussion

Since the routine use of neoadjuvant chemotherapy began in the 1980s, limb salvage surgery reconstruction is generally the favored treatment option when managing extensive primary bone tumours, with limb salvage rates above 90% in most major centers. Endoprostheses have several advantages over biological reconstruction methods, being readily available in both custom-made and modular forms, initially reliable with low complication rates, and allowing rapid return to full weight bearing with predictable function [5].

Endoprosthetic replacement following tumor resection is a good treatment option for proximal femoral malignant lesions, due to its low complication rate and good postoperative function [4, 6]; however, the management of a proximal femoral malignant lesion with intra-articular involvement is challenging, especially in pediatric patients. The resection of a tumor at the level of the proximal femur results in loss of abductors and other musculature necessary for hip stability. This often leads to a higher dislocation rate. Hip dislocation is a recognized problem after the use of megaprostheses, with rates of dislocation varying from 1.7% to around 28% [4, 7, 8]. To reduce the risk of dislocation in our case, we decided to use a bipolar hemiarthroplasty as it is inherently more stable than total hip replacement. We reattached the abductors to a surgical mesh and directly to the prosthesis attachment site.

Periacetabular en bloc resection of the pelvis (Enneking [9] type II resection) including the anterior and posterior columns is the traditional treatment for malignant bone lesions affecting the hip joint, but this option may result in substantial loss of pelvic bone stock and compromise pelvic stability. Although several other reconstructive procedures have been reported, including pelvic prosthesis arthroplasty [10–14], allograft reconstruction (with or without a total hip prosthesis) [15, 16] arthrodesis [13, 17], and pseudarthrosis [13], a gold standard has yet to be established owing to poor postoperative function and major complication rates ranging from 33% to 56%.

Rüdiger et al. [18] described a posterior-column-preserving technique for the treatment of intra-articular malignant lesions, using the Bernese periacetabular osteotomy introduced in the 1980s by Ganz et al. [19] for the treatment of hip dysplasia. They reported two cases with intra-articular extension of a proximal femoral malignant lesion treated with extra-articular resection of the hip, reconstructed with a structural acetabular allograft and endoprosthetic replacement maintaining the continuity and stability of the pelvic ring. The use of the Bernese periacetabular osteotomy in our case had the main advantage of preserving the stability of the pelvic ring and also preserving a large bone contact area with intact vascular supply from the superior gluteal artery to the supra-acetabular and posterior column bone [20, 21]. Both facts contributed to early consolidation/integration of the irradiated graft and allowed increased loading of the hip and range-of-motion training [22].

Extracorporeal irradiation of autogenous timorous bone and its use for reimplantation was first described in 1968, by Spira and Lubin [23]. Since then, extracorporeal irradiation and reimplantation (ECI) has been used as a surgical limb salvage technique for musculoskeletal malignant lesions. The major advantages of this surgical technique are biological reconstruction, no risk of disease transmission or immunological reaction, ready availability, and preservation of bone stock [24]. A recently published case series of 30 malignant tumours treated with extracorporeal irradiation and posterior reimplantation reported 90% excellent and good results, with two cases of surgical failure, and a 10% infection rate as the main complication [24]. In our case, although no evidence of tumor extension into the acetabulum was seen on MRI, there was extension into the capsule joint due to the pathological neck fracture. For this reason we considered the tumor as intra-articular and performed an extra-articular resection of the hip joint, in order to secure wide excision margins. Taking into account that we were treating a skeletally immature patient, we decided to reimplant the resected acetabulum after irradiation, in order to guarantee a perfect geometric match and because we did not have access to an acetabulum allograft of pediatric size.

The irradiation of the autologous acetabular graft in a skeletally immature patient may result in a premature fusion of the triradiate physeal cartilage. Fusion of the triradiate cartilage, as seen in young patients with pelvic or acetabular fractures, produces a disparate growth between the femoral head and the acetabulum, with subsequent incongruence of the hip joint resulting in subluxation of the femoral head, similar to the mechanism in developmental dysplasia of the hip [25]. In our case, although we reimplanted the autologous acetabular graft after extracorporeal irradiation, we replaced the proximal femur with a megaprosthesis, which should avoid future incongruence or mismatch between the prosthetic bipolar head and the acetabulum.

We conclude that an extra-articular resection of the hip using the Bernese periacetabular technique, followed by reconstruction with an extracorporeally irradiated acetabular autograft and a proximal femur modular megaprosthesis, is a useful combined treatment option for malignant femoral lesions involving the hip joint, especially in pediatric patients.
